# Canine Urothelial DNA Mutations Are Not Associated With Total Trihalomethanes Concentrations in Municipal Drinking Water

**DOI:** 10.1111/vco.70060

**Published:** 2026-03-12

**Authors:** Janice O'Brien, Samantha L. Braman, Jennifer Willcox, Phillip Wang, Daniel Gerrity, Audrey Ruple, Lauren A. Trepanier

**Affiliations:** ^1^ Department of Population Health Sciences Virginia‐Maryland College of Veterinary Medicine Blacksburg Virginia USA; ^2^ Department of Medical Sciences, School of Veterinary Medicine University of Wisconsin‐Madison Madison Wisconsin USA; ^3^ Antech Diagnostics, Mars Petcare Science & Diagnostics Loveland Colorado USA; ^4^ Southern Nevada Water Authority Las Vegas Nevada USA

## Abstract

Contaminants in municipal drinking water, specifically total trihalomethanes (TTHMs), are mutagenic. Exposures to TTHMs have been associated with urothelial carcinoma (UC) in both people and dogs. Mutations in the *BRAF* gene and other genomic copy number aberrations detected in voided urothelial cells with commercial CADET BRAF and BRAF‐PLUS tests have also been associated with the presence of UC in dogs. However, it is not known whether these urothelial mutations can be linked to TTHM exposures in dogs. The objectives of this ecological study were to compare the incidence of detected urinary *BRAF* mutations or genomic copy number aberrations in dogs residing in a city with relatively high drinking water TTHMs (Las Vegas, NV) to a city with significantly lower TTHMs (Reno, NV), and to expand the study group to include BRAF and BRAF‐PLUS test results and municipal drinking water TTHM concentrations by zip code across the United States. Dogs living in Las Vegas had a higher relative risk of urothelial mutations compared to dogs living in Reno (RR 2.52; 95% CI, 1.77–3.38; *p* < 0.0001). However, this risk could not be attributed to higher municipal drinking water TTHMs. Across the United States, the population‐adjusted incidence of urothelial mutations in voided urine was not associated with municipal water TTHMs, but was instead associated in cross‐sectional analyses with age, neutered status, higher regional test submission rates and previously reported high‐risk breeds for UC (Scottish terriers, West Highland white terriers, Shetland sheepdogs, beagles and wirehaired fox terriers). The higher RR for a positive BRAF or BRAF‐PLUS test in Las Vegas versus Reno could be solely due to higher test submission rates (2.97 versus 0.97 per 1000 dogs) but could also reflect other environmental exposures not considered in this study.

## 1 Introduction

Urothelial carcinoma (UC) in dogs is typically an invasive cancer that can lead to haematuria, dysuria, secondary infections and urethral obstruction, and is often life‐limiting [1]. *BRAF* ^*V595E*^ is a somatic mutation that is detected in 59%–87% of canine UC tissue samples [2, 3, 4, 5] and in 83% of voided urine samples in dogs with confirmed UC [6]. Copy number and other chromosomal variants can also be detected in the urine of dogs with UC with the CADET BRAF‐PLUS assay, which further increases sensitivity for urothelial mutations [7]. Urine testing for these variants has allowed for less invasive, less expensive and earlier screening for UC risk [8]. Understanding risk factors for these acquired somatic variants is important to develop better prevention strategies for UC in dogs.

UC is associated with environmental exposures in both dogs and humans. Smoking is a major risk factor for UC in people [9], but studies evaluating second hand smoke in dogs have found mixed results [10, 11, 12, 13]. However, contaminants in municipal drinking water, including disinfection byproducts and specifically trihalomethanes (THMs), have been linked to UC in both people [14] and dogs [10, 15]. THMs are mutagenic byproducts of water disinfection, especially chlorination [16]. In public water systems, the United States Environmental Protection Agency (EPA) regulates the combined concentration of four THMs, chloroform, bromodichloromethane, dibromochloromethane and bromoform, which are described collectively as total trihalomethanes (TTHMs).

Because canine UC was associated with municipal drinking water TTHM concentrations in two separate case‐control studies [10, 15], we wanted to test the hypothesis that dogs living in areas with relatively high municipal water concentrations of TTHMs would have a higher incidence of *BRAF* ^*V595E*^ or other urothelial genomic variants compared to dogs living in areas with lower municipal water TTHM concentrations. The first objective of this ecologic study was to compare the incidence of positive CADET BRAF or BRAF‐PLUS variants in voided urothelial cells from dogs residing in two cities with distinctly different drinking water TTHM concentrations: Las Vegas and Reno Nevada. The second objective was to expand the study group to evaluate these urothelial DNA mutations and municipal drinking water TTHM concentrations by zip code across the United States based on submissions to a national commercial diagnostic laboratory.

## 2 Methods and Materials

### 2.1 TTHM Concentrations by Zip Code

For Nevada, municipal drinking water TTHM concentrations were provided for Las Vegas by the Southern Nevada Water Authority and for Reno by the Truckee Meadows Water Authority. Data were originally generated for regulatory compliance purposes and were provided by zip code from January 2017 through December 2023. For this study, concentrations were then averaged for each zip code over the 7‐year period.

Across the United States, municipal drinking water TTHM concentrations were obtained from the Environmental Working Group (EWG) database (https://www.ewg.org/tapwater/). Data were provided as a single average value from 2014 to 2023, or as available. For zip codes with more than one municipal water authority, TTHM concentrations for the primary (largest) water authority were recorded.

### 2.2 BRAF Test Results

Results from canine urine samples submitted for detection of *BRAF* ^*V595E*^ (CADET BRAF) were provided by a single commercial laboratory (Antech Diagnostics, Mars Petcare Science & Diagnostics) for the 5‐year period from January 2019 (when the laboratory started offering the assay) through December 2023. Some clinical samples that were negative for *BRAF* ^*V595E*^ were further screened for other genomic copy number aberrations by droplet digital PCR (the CADET BRAF‐PLUS assay) [7]; these additional variants have also been associated with canine UC. Including additional genomic variants increases the overall assay sensitivity for canine UC [17], although the overall sensitivity for CADET BRAF‐PLUS has not been fully evaluated.

All tests submitted for BRAF testing to Antech Diagnostics during the 5‐year observation period were sorted by zip code. For the first aim, analyses were focused on zip codes serviced by the Southern Nevada Water Authority (Las Vegas) and the Truckee Meadows Water Authority (Reno). For the second aim, the search was expanded to include BRAF tests submitted from any U.S. zip code for the same 5‐year period. Samples from Canada and U.S. territories were excluded from analyses, since matching TTHM data were not available. Submitted canine urine samples that did not return a BRAF result were also censored. If multiple urine samples were submitted from the same dog, only the first submission was included in analyses.

### 2.3 Estimated Dog Populations

Calculating the incidence of positive urothelial mutation tests over time required estimates of total pet dog populations. The estimated dog population for each zip code was derived from the 2020 U.S. Census Bureau household numbers [18], along with American Veterinary Medical Association (AVMA) surveys of the average percentage of households owning dogs (44.6%) and average dogs per household (1.46) [19].

### 2.4 Statistical Analyses

For the first objective, median municipal drinking water TTHM concentrations across all zip codes from 2017 to 2023 were compared between Las Vegas and Reno using a Mann‐Whitney U test. The incidence of urothelial DNA mutation detection was calculated as the total number of positive CADET BRAF or BRAF‐PLUS test results by zip code over the observation period, divided by the estimated dog population by zip code. This was used to calculate the relative risk for urothelial mutation detection in Las Vegas versus Reno, with 95% confidence intervals (CI).

Unconditional logistic regression was then used in cross‐sectional analyses with area‐level exposure assignment to generate odds ratios for urothelial mutation detection relative to municipal drinking water TTHM concentrations, age, sex, neuter status and high‐risk breeds (Scottish terrier, West Highland white terrier, Shetland sheepdog, beagle or wirehaired fox terrier) [20] versus other breeds. A similar approach was used for the second objective (nationwide analysis), except that municipal drinking water TTHM concentrations by zip code across the United States were obtained from the EWG database. In addition, the incidence of urothelial DNA mutation detection was modelled for zip codes with average TTHMs over the observation period in three categories (< 50 μg/L, 50–100 μg/L and > 100 μg/L) [21]. Unfortunately, environmental tobacco smoke exposures were not available to include in the models.

Finally, logistic regression was also used to assess whether positive BRAF or BRAF‐PLUS test results were associated with regional testing rates, adjusted for estimated dog populations across the country. All regressions were performed using the glm() function in R version 4.4.2. Formulas for both regression models are shown in Appendix A.

## 3 Results

### 3.1 TTHM Concentrations by Nevada Zip Codes

Total trihalomethane concentrations in municipal drinking water over 7 years were significantly higher in Las Vegas zip codes (median 50.7 μg/L, observed range 2–84 μg/L) compared to Reno zip codes (median 27.0 μg/L, observed range 0–75 μg/L; *p* < 0.0001; Table 1). However, both systems were compliant with U.S. EPA regulations for TTHM concentrations, with locational running annual averages below 80 ug/L.

**TABLE 1:** Total trihalomethane (TTHM) concentrations in municipal water for zip codes in Las Vegas NV, measured by the Southern Nevada Water Authority and in Reno NV, measured by the Truckee Meadow Water Authority. Data shown are medians and observed ranges for 2017 through 2023.

Las Vegas zip code^a^	TTHMs (μg/L or ppb)	Reno zip code	TTHMs (μg/L or ppb)
89 108	44.9 (2–62)	89 436	30.5 (20–57)
89 129	57.0 (43–76)	89 441	29.0 (20–54)
89 134	57.5 (44–84)	89 502	33.5 (14–54)
89 135	51.0 (34–69)	89 503	27.5 (16–42)
89 138	52.4 (40–74)	89 506	7.5 (2–53)
89 141	48.0 (29–70)	89 519	18.0 (10–41)
89 143	61.1 (24–73)	89 521	26.5 (12–64)
89 144	51.3 (37–63)	89 523	31.0 (0–75)
89 147	43.5 (37–51)	89 511	27.0 (0–61)
89 148	46.7 (38–59)		
89 166	53.0 (32–70)		
89 178	44.3 (38–51)		
All zip codes	50.7^a^ (2–84)		27.0 (0–75)

^a^ *p* < 0.0001.

### 3.2 BRAF Test Results by Nevada Zip Code

Study group demographics for dogs with detected urothelial DNA variants in Las Vegas (*n* = 143 positive dogs) and Reno (*n* = 40 positive dogs) are shown in Table 2. The relative risk for a submitted positive *BRAF* ^*V595E*^ or genomic copy number variant test result for dogs living in Las Vegas versus Reno was 2.52 (95% CI, 1.77–3.38; *p* < 0.0001; Table 3).

**TABLE 2:** Demographic characteristic of pet dogs from Las Vegas and Reno NV testing positive for *BRAF* ^*V595E*^ (CADET BRAF test) or genomic copy number variants (CADET BRAF‐PLUS test) in voided urine samples submitted to a national diagnostic laboratory during the 5‐year period from 2019 to 2023.

	Las Vegas	Reno
Positive urothelial mutation(s) detected	*n* = 143 dogs	*n* = 40 dogs
Median age	11.0 years	10.8 years
Range	(2–17)	(6–15)
Sex/neuter status^a^	FS *n* = 70 (49.2%) MN *n* = 68 (47.9%) MI *n* = 3 (2.1%) FI *n* = 1 (0.7%)	FS *n* = 22 (56.4%) MN *n* = 14 (35.9%) MI *n* = 2 (5.1%) FI *n* = 1 (2.6%)
Breeds represented more than once	Mixed *n* = 25 Not provided *n* = 31 Dachshund *n* = 6 Am Staff terr *n* = 6 Beagle *n* = 6 Pomeranian *n* = 6 Lab retriever *n* = 5 Chihuahua *n* = 4 Germ shep *n* = 4 Maltese *n* = 4 Min pinsch *n* = 4 York terr *n* = 4 Shih tzu *n* = 3 Am Eskimo *n* = 3 Min poodle *n* = 2 Shetl sheep *n* = 2 Staff bull terr *n* = 2	Mixed *n* = 13 Not provided *n* = 5 Austr shep *n* = 2 Dachshund *n* = 2 Lab retriever *n* = 2 Min pinsch *n* = 2

^a^ Sex/neuter status was not provided for 1 dog each from Las Vegas and Reno.

**TABLE 3:** Relative risk for a positive *BRAF* ^*V595E*^ mutation (CADET BRAF assay) or genomic copy number aberrations (CADET BRAF‐PLUS assay when performed), in urine from pet dogs in Las Vegas and Reno, NV. Positive results are associated with urothelial carcinoma in dogs. Urine assays were performed at Antech Diagnostics (Mars Petcare Science & Diagnostics) from 2019 through 2023.

City	Estimated total dog population in included zip codes^a^	CADET tests performed	Overall positive results	% positive by tests performed	Estimated incidence (per 1000 dogs)	Relative risk (95% CI)
Las Vegas	93 743	*n* = 278	*n* = 143	51.4%	1.523	2.71
Reno	71 071	*n* = 69	*n* = 40	58.0%	0.563	(1.91–3.84)^b^

^a^ Estimated from the 2020 U.S. household census and AVMA data on average dog ownership per household.

^b^ *p* < 0.0001 relative to Reno NV zip codes.

The odds of positive urothelial DNA variants in submitted canine urine samples for Las Vegas and Reno are summarised in Table 4. Urothelial mutation detection was not associated with municipal water TTHMs (OR 0.69, 95% CI 0.39–1.21, *p* = 0.20) in the Nevada dataset. However, it was associated with greater age (OR 1.15, 95% CI 1.07–1.25; *p* < 0.001).

**TABLE 4:** Odds ratios for a positive urinary *BRAF* ^*V595E*^ mutation (CADET BRAF assay) or genomic copy number aberrations (CADET BRAF‐PLUS assay when performed), per estimated 1000 pet dogs per zip code in Las Vegas versus Reno Nevada.

Status	Odds ratio for urothelial mutation detection	*p*	Lower 95% CI	Higher 95% CI
Las Vegas versus Reno	0.69	0.20	0.39	1.21
Age	1.15	< 0.001	1.07	1.25
Female sex	0.77	0.25	0.49	1.21
Neutered status	2.60	0.06	0.99	7.36
Predisposed breed^a^	1.46	0.44	0.57	4.08

^a^ Scottish terrier, West Highland white terrier, Shetland sheepdog, beagle or wirehaired fox terrier [20].

### 3.3 BRAF Test Results and TTHMs by U.S. Zip Code

Urinary BRAF test results were returned by Antech Diagnostics to U.S. veterinarians for 67 984 canine urine samples for the 5 years from 2019 through 2023, excluding samples from U.S. territories.

Total trihalomethane data were available for 6634 of 7508 U.S. zip codes (88.4%) from which samples were submitted for BRAF testing; municipal drinking water TTHM data were not available on the EWG database for any zip codes in the state of New Hampshire. The final number of urine samples with corresponding TTHM data was 64 144 (94.4%) of BRAF laboratory results provided within the study period. Across the United States, the estimated incidence of positive tests was not significantly associated with municipal drinking water TTHM concentrations (OR 1.00, 95% CI, 1.00–1.00; *p* = 0.63, Table 5) in the ecologic study design by zip code. However, BRAF or BRAF‐PLUS mutation detection in this national dataset was positively associated in cross‐sectional analyses with age, neutered status and predisposed breeds (*p* < 0.0001), while female sex had an unexpectedly lower odds ratio for *BRAF* mutation detection (Table 5).

**TABLE 5:** Odds ratios for a positive urinary *BRAF* ^*V595E*^ mutation or genomic copy number aberration (CADET BRAF‐PLUS test), per estimated 1000 pet dogs per zip code, in voided canine urine samples submitted to national diagnostic laboratory from 2019 through 2023 across the U.S. TTHM: Total trihalomethane concentrations in municipal drinking water.

Status	Odds ratio for urothelial mutation detection	*p*	Lower 95% CI	Higher 95% CI
TTHM concentrations	1.00	0.63	1.00	1.00
Age	1.18	< 0.0001	1.18	1.19
Female sex	0.75	< 0.0001	0.72	0.77
Neutered status	2.54	< 0.0001	2.37	2.71
Predisposed breed^a^	2.65	< 0.0001	2.47	2.85

^a^ Scottish terrier, Scottish terrier, West Highland white terrier, Shetland sheepdog, beagle or wirehaired fox terrier [20].

When municipal water TTHMs were binned into three groups, the adjusted odds ratios were 0.99 (95% CI 0.95–1.04, *p* = 0.42) for the 50–100 μg/L group and 0.81 (95% CI 0.0.48–1.34, *p* = 0.58) for the > 100 μg/L group, each compared to the < 50 μg/Lreference group. The remainder of the estimates for age, sex, neuter status and predisposed breed with this binned model were consistent to two decimal places with the values for the primary regression model shown in Table 5.

We also assessed whether different regional testing rates (per estimated 1000 dogs per zip code) could bias towards higher urothelial DNA mutation detection rates. Indeed, higher testing rates were associated with a higher odds ratio for a positive test result (OR 1.31, 95% CI, 1.31–1‐32, *p* < 0.0001) across the U.S. sampling group. For context, Las Vegas had a testing rate of 2.97 per 1000 dogs and Reno had a testing rate of 0.97 per 1000 dogs.

## 4 Discussion

Of the Nevada zip codes included in this ecological study, the chlorinated municipal drinking water in Las Vegas had nearly a 2‐fold higher TTHM concentration compared to the chlorinated municipal drinking water in Reno. The Las Vegas water supply is primarily sourced from Lake Mead within the Colorado River system. Las Vegas' desert climate creates warmer water temperatures within the drinking water distribution system, which leads to greater TTHM formation [22]. In contrast, the Reno water supply is primarily sourced from the Truckee River, which is fed by snowmelt from the Sierra Nevada Mountains via Lake Tahoe. Relative to Las Vegas, this leads to lower TTHM formation.

We found that dogs living in these Las Vegas zip codes had a higher relative risk of a positive BRAF or BRAF‐PLUS test result compared to dogs living in the Reno zip codes. However, mutation detection was not directly associated with municipal drinking water TTHM concentrations in the Nevada dataset, suggesting that the higher relative risk in Las Vegas was not explained by this water quality parameter. Across the larger national dataset, we found that higher testing rates were associated with a higher odds ratio of a positive test. Thus, the higher relative risk for urothelial mutation detection in Las Vegas could simply be due to the higher per capita testing rate. It could also be due to differences in other environmental exposures not considered in this study. Although data were not available at the zip code level, tobacco use rates appear similar between Las Vegas and Reno (15.5% versus 15.0%, respectively), based on a 2022 census (places.cdc.gov).

We also did not find an association between urothelial DNA mutation detection and municipal drinking water TTHM concentrations in samples submitted to Antech Diagnostics across the United States in the 5‐year period from 2019 to 2023. This contrasts with two separate prospective case‐control studies, which found that dogs with UC resided in counties with an average of 3‐ to 4‐fold higher municipal water TTHM concentrations compared to unaffected control dogs [10, 15]. Our ecologic study design was not structured to evaluate a direct association between TTHMs and UC, since we did not have data to support a definitive UC diagnosis for the submitted urine samples.

It is possible that TTHMs associate with UC in dogs through different mechanism(s) than the tested urothelial mutations. However, an earlier study found no significant differences in estimated exposure to TTHMs between dogs with UC and controls [23]. This earlier study did not match cases and controls but did estimate TTHM exposures over time from drinking water utility data and the dogs' owner‐reported residence histories. They also included individual data on household smoking and whether dogs drank household tap water, and these data were missing from the two studies with positive TTHM associations.

In human patients, municipal drinking water TTHM concentrations have been linked to bladder cancer in several studies [21, 24, 25]. In addition to drinking, exposures to TTHMs can occur during bathing or swimming [26]. In fact, swimming in a chlorinated pool was associated with DNA damage (micronuclei in peripheral blood) in healthy adults, which was correlated with exhaled concentrations of three brominated halomethanes [27]. Swimming in a pool was also associated with UC in a small case‐control study in dogs [10]. Unfortunately, we did not have data in the current canine study on bathing frequency or swimming pool use.

Our study has important limitations. While we had a relatively large sample size of dogs with submitted BRAF testing and available municipal drinking water TTHM concentrations, TTHM data were not available for all zip codes and were completely missing for the state of New Hampshire.

In addition, the detection of the *BRAF* ^*V595E*^ mutation or genomic copy number variants is not diagnostic of UC in dogs without additional information such as clinical signs, bladder imaging and histopathology, the gold standard for UC diagnosis [13]. The absence of *BRAF* ^*V595E*^ does not rule out canine UC [28], and not all *BRAF* ^*V595E*^ undetected urine samples in the current study were further tested for other genomic variants because of inadequate urine volume remaining. In addition, *BRAF* ^*V595E*^ is not specific to UC of the bladder and urethra and can be found in the urine of some dogs with prostatic carcinoma, which can have a variety of cellular origins [5], including urothelial cells with or without bladder involvement [29]. This could have biased the odds ratio in favour of male dogs in the current study, depending on the number of prostatic carcinoma cases submitted.

Our population denominators were based on estimated dogs per household in each zip code, and we were unable to age‐adjust reference populations based on available data. While this limitation applies across zip codes, it is possible that some zip codes had more elderly dogs at risk in the reference population than others. Furthermore, we had no zip code‐level data on environmental tobacco smoke exposures or other environmental risk factors for UC.

Another important limitation is that we did not have individual‐level data on whether dogs were provided bottled, filtered or well water instead of municipal drinking water. Dogs with these other drinking water sources would not be at risk from drinking water TTHMs. The use of well or bottled drinking water could also vary by zip code and socioeconomic status. We also had no data on swimming and bathing habits; inhalation and dermal absorption during showering, swimming and bathing are also important routes of TTHM exposures in people [24, 30]. Finally, the potential lag times between chemical exposures and canine UC are not understood, and we did not have access to residence histories for these dogs to estimate lifetime TTHM exposures.

## 5 Conclusion

These results, based on local data (Las Vegas and Reno, NV) and across a national sample of canine urine samples submitted for BRAF testing, do not support a relationship between municipal drinking water TTHMs and urothelial DNA mutations detected using commercially available assays. At the national level, positive test results were significantly associated, as expected, with age, neutered status and previously reported high‐risk breeds, as well as higher regional testing rates.

## Funding

We thank Mr. Duncan Alexander for a gift in support of this study.

## Ethics Statement

BRAF results by zip code were provided by Antech Diagnostics and were individually de‐identified.

## Conflicts of Interest

J.W. is currently an employee of Antech Diagnostics (Mars Petcare Science & Diagnostics). CADET BRAF and BRAF‐PLUS are products of Antech Diagnostics (Mars Petcare Science & Diagnostics).

## Data Availability

The data that support the findings of this study are available from the corresponding author upon reasonable request.

## Appendix A

The logistic regression formula used to model BRAF mutation detection, TTHMs, age, sex, neuter status and breed for Nevada (Las Vegas and Reno) and U.S. zip codes:

$$\log\frac{P\left(\text{BRAF}{+}_{i}\right)}{1-P\left(\text{BRAF}{+}_{i}\right)}=\beta_{0}+\beta_{1}{\text{TTHM}}_{i}+\beta_{2}{\mathrm{Age}}_{i}+\beta_{3}{\mathrm{Sex}}_{i}+\beta_{4}{\text{Neuter}}_{i}+\beta_{5}{\text{PredBreed}}_{i}$$

where $\text{BRAF}{+}_{i}$ was the positive BRAF mutation detection status of the individual test for dog i, $\exp\left(\beta_{1}\right)$ was the adjusted odds ratio for a 1 μg/Lincrease in municipal drinking water TTHM (total trihalomethane) concentrations for that zip code, $\exp\left(\beta_{2}\right)$ was the adjusted odds ratio for a 1‐year increase in age, $\exp\left(\beta_{3}\right)$ was the adjusted odds ratio for females (males as reference), $\exp\left(\beta_{4}\right)$ was the adjusted odds ratio for neutered dogs (intact as reference) and $\exp\left(\beta_{5}\right)$ was the adjusted odds ratio for predisposed breeds (non‐predisposed breeds as reference).

The logistic regression formula used to model BRAF mutation detection by TTHM concentrations and testing rates:

$$Y_{j}=\beta_{0}+\beta_{1}\;{\text{TTHM}}_{j}+\beta_{2}\;{\text{TestingRate}}_{j}$$

where $\beta_{1}$ is the change in BRAF mutations detected per 1000 dogs for each 1 μg/L increase in TTHM, adjusting for testing rate; $\beta_{2}$ is the change in BRAF mutations detected per 1000 dogs for each additional test per 1000 dogs, adjusting for TTHM concentration and $Y_{j}$ is the number of positive tests per 1000 dogs in zip code *j*.
